# Design Optimization of Reconfigurable Liquid Crystal Patch Antenna

**DOI:** 10.3390/ma14040932

**Published:** 2021-02-16

**Authors:** Dowon Kim, Kitae Kim, Hogyeong Kim, Moonyoung Choi, Jun-Hee Na

**Affiliations:** 1 Department of Electrical, Electronics & Communication Engineering Education, Chungnam National University, Daejeon 34134, Korea; dowonk@cnu.ac.kr (D.K.); hg1105@o.cnu.ac.kr (H.K.); 2 Department of Convergence System Engineering, Chungnam National University, Daejeon 34134, Korea; kitaekim@o.cnu.ac.kr (K.K.); 201603516@o.cnu.ac.kr (M.C.)

**Keywords:** liquid crystal, dielectric anisotropy, frequency tunable antenna, smart antenna

## Abstract

In various fields such as the 5G antenna system and satellite communication system, there is a growing demand to develop a smart antenna with a frequency selective or beamforming function within a limited space. While antennas utilizing mechanical, electronic, and material characteristics are being studied, a method of having tunable frequency characteristics by applying a liquid crystal material with dielectric anisotropy to a planar patch antenna is proposed. In resonance mode, the design method for using only the minimum amount of expensive liquid crystals is systematically arranged while maximizing the amount of change in the operating frequency of the antenna by considering the electric field distribution on the surface of the patch antenna. Furthermore, to increase the dielectric anisotropy of the liquid crystal, the liquid crystal must be aligned. Simultaneously, in cases where the cell gap of the liquid crystal exceeds 100 μm, the alignment force is weakened. While compensating for this shortcoming, securing the radiation characteristics of the antenna is proposed, and simulations are performed.

## 1. Introduction

Research on a multi-functional smart antenna is actively conducted. Systems that can quickly exchange large amounts of information, such as satellite communications, next-generation mobile networks, and radar systems, are required [1,2]. At the same time, the size of the hardware is increasingly miniaturized and flattened. Therefore, it can be said that the development of an antenna for transmitting and receiving broadband information while covering various frequency bands in a limited space is an important task [3,4,5,6].

Accordingly, various studies have been performed on an active frequency-selective antenna to cover various application frequencies simultaneously. There is research of selectively using frequency by changing the size of the antenna mechanically [7,8,9,10], electronically frequency tunable antennas using semiconductor devices such as MEMS [11,12], and research of utilizing variations of material properties such as ferroelectric or ferrite [13,14,15].

Here, we propose applying a liquid crystal to a frequency tunable patch antenna for a microwave frequency band. This liquid crystal is being actively studied in the field of an antenna for variable frequency [16,17,18,19,20] and an electronic beamforming antenna for the mm-wave frequency band [21,22,23,24,25,26]. However, research on technology for securing antenna performance while using an expensive liquid crystal to a minimum is still insufficient [27]. In particular, we show a method of maximizing the antenna frequency change rate while using only the minimum amount of expensive liquid crystal. Considering the electric-field distribution on the surface of the patch antenna and the polarization ratio inside the liquid crystal according to the thickness of the antenna substrate, a design method that optimizes the injection position and quantity of liquid crystals in the directions parallel and perpendicular to the antenna surface was developed.

## 2. Materials and Methods

### 2.1. Modeling of Numerical Calculation

**Figure 1:**
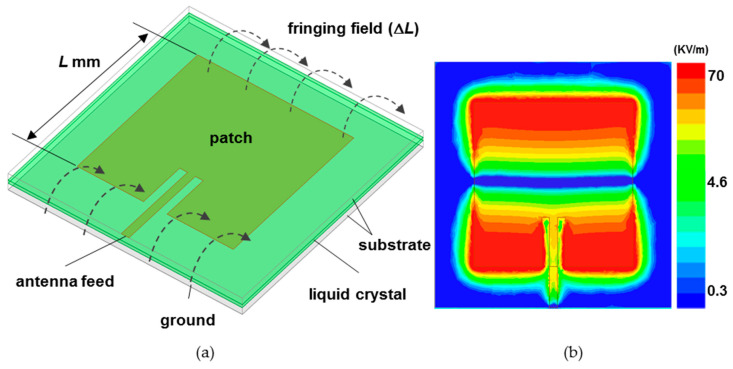
(**a**) Schematic diagram of a tunable liquid crystal patch antenna and (**b**) electric-field distribution on a patch antenna surface. Here, *L* and ∆*L* refer to the patch length and fringing field length, respectively.

A patch antenna is a planar shape that induces electromagnetic radiation through the gap between sandwiched metallic plates on top and bottom planes. As shown in Figure 1a, the total length of the patch length (*L*) and fringing field length (∆*L*) parallel to the wave propagation direction generates resonance at a frequency corresponding to the half wavelength, causing the radio waves to radiate into the air. If this is expressed more clearly as a formula [28], the resonance frequency (*F*_res_) can be calculated as *F*_res_ = *c*/(2*L*_e_√*ε*_e_), *L*_e_ = *L* + 2*L*. The *c* is the speed of light, and the *ε*_e_ is the effective permittivity of the substrate. The factors that determine the resonant frequency (*F*_res_) are the patch antenna length and substrate thickness and the dielectric constant of the substrate material. By replacing the substrate with a liquid crystal, it is possible to implement a frequency tunable patch antenna by utilizing the dielectric anisotropy of the liquid crystal according to the voltage.

In resonance mode, the electric field on the surface of the patch antenna is strongly coupled at both ends of the direction of the wave propagation as shown in Figure 1b, and null in the center region. Therefore, focusing on this electric-field distribution, we systematically analyze the effect of dielectric anisotropy of the liquid crystal on the operation of the patch antenna while controlling the injection location and the area of the liquid crystal. Additionally, in the case of ferrite, ferroelectric, and semiconductor devices, the dielectric loss increases as the frequency increases, whereas the dielectric loss of liquid crystal decreases as the frequency increases [29,30,31]. So, it is advantageous when used as a tunable device in the mm-wave frequencies. However, since liquid crystal is a costly material and cannot be used indefinitely, research to maximize tunability while using a minimum amount of liquid crystal is necessary. First, the position and the area of the liquid crystal on the surface of the patch, which significantly affects the amount of frequency change, are determined by considering the electric-field distribution over the antenna surface. Second, in terms of the substrate thickness, the effect of the thickness of the liquid crystal layer on the frequency change rate is analyzed, and finally, an idea for maximizing the frequency change rate and securing the radiation performance of the antenna is proposed. It is expected to provide a design rule for optimizing the performance of a patch antenna for variable frequency using a minimum amount of liquid crystal. In this paper, all the simulations are performed by using an HFSS (high-frequency structure simulator, Ansys Inc.), which is a tool for analyzing electromagnetic wave propagation in the microwave frequencies.

### 2.2. Liquid Crystal

**Figure 2:**
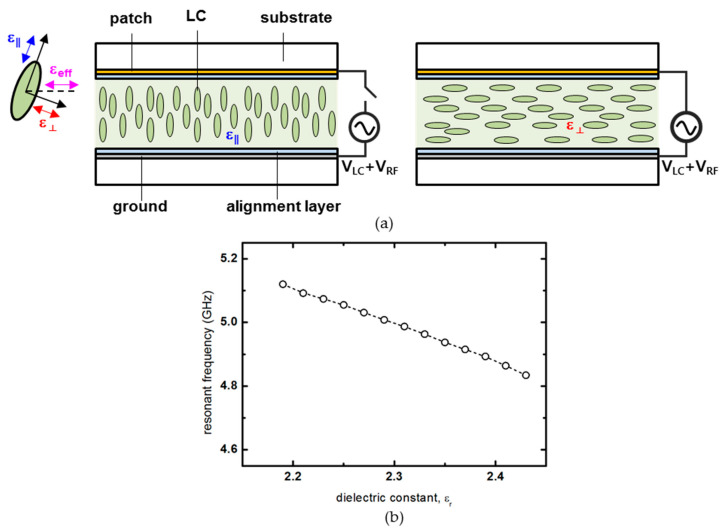
(**a**) Schematic diagram of dielectric anisotropy according to the voltage applied to the vertically aligned liquid crystal used as the substrate of the patch antenna. (**b**) Resonant frequency change according to a dielectric constant of liquid crystal.

Liquid crystal is a material having dielectric constant anisotropy, in which the dielectric constant changes according to the movement of the polarization of liquid crystal molecules with the applied voltage. The liquid crystal used here is a nematic phase liquid crystal, having directional ordering, with a vertically aligned configuration. When the voltage is not applied, the polarization stands vertically on the surface of the alignment layer. They can be described in the form of a rod, as shown in Figure 2a. When the elongated polarization direction is parallel to the electric field direction, the permittivity is *ε*_∥_. When the elongated polarization direction is perpendicular to the electric field direction, the permittivity corresponds to *ε*_⊥_. The value of this permittivity continuously changes from *ε*_∥_ to *ε*_⊥_ according to the applied voltage, and this change in permittivity makes it possible to continuously change the resonant frequency of the patch antenna in Figure 2b. The estimated dielectric constants *ε*_∥_ and *ε*_⊥_ calculated from the refractive index values of the used liquid crystal are 2.19 and 2.43, respectively. Here, we propose a core model for patch antenna having a variable dielectric constant through the behavior of liquid crystal molecules that are changed by an external electric field. Therefore, V_LC_, which affects the behavior of the liquid crystal, and V_RF_, which is the signal component of the antenna, are applied to the liquid crystal cell, as shown in Figure 2a. According to the dielectric constant of the nematic phase liquid crystal used in this study, the resonance frequency is ~0.3 GHz. The liquid crystal used in this numerical calculation used Merck’s liquid crystal with a negative dielectric constant. The material parameters of the liquid crystal were optical anisotropy n = 0.1091, isotropic temperature 108 °C, splay elastic constant K_11_ = 13.8 pJ/m, and bend elastic constant K_33_ = 18.1 pJ/m.

## 3. Results and Discussion

### 3.1. Analysis of Optimal Liquid crystal Position on the Patch Surface

As mentioned in the previous chapter, the operating frequency of the patch antenna is determined according to the patch length in the wave propagation direction, and radiation mainly occurs at both ends of the wave propagation direction. Therefore, considering the electric field distribution on the surface, the analysis is conducted through simulation to see how the frequency change rate changes while partially injecting a liquid crystal. In the nematic phase LC with directional ordering, the LC director can be controlled by the electric field, and dielectric constant change can be generated through the direction of LC director. In this simulation, the frequency change was confirmed by varying the dielectric constant in the electric field applied through the upper and lower patch antenna electrodes.

In Figure 1a, glass substrates with a dielectric constant of 5.5 are used as the substrates with the patch antenna and the metal ground plane, and the patch antenna is placed on top of the upper glass substrate and the metal ground on the bottom of the lower glass substrate. The thickness of the upper and lower glass substrates is fixed at 100 μm each. The liquid crystal substrate is inserted into the center between the two glass substrates as the part marked green in Figure 1a, and the thickness of the liquid crystal layer is also fixed at 100 μm. The size of the patch antenna is designed to operate in the 3.5 GHz band with a size of 20 × 20 mm^2^, and the size of the metal ground plane is fixed at 30 × 30 mm^2^.

**Figure 3:**
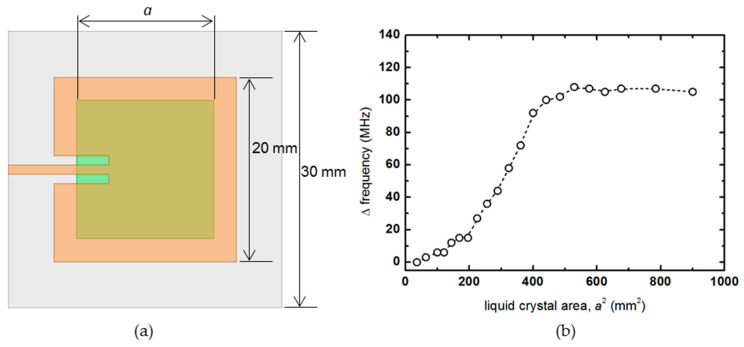
(**a**) Schematic diagram of the patch antenna that increases the liquid crystal area in a square shape, and (**b**) the amount of frequency change.

In the first analysis process, the liquid crystal area is set as an independent variable, as shown in green in Figure 3a. The thickness of the liquid crystal is fixed at 100 μm, and a square liquid crystal is placed in the center of the patch antenna, and the length of one side is gradually increased to calculate the rate of frequency change for the area *a* × *a* mm^2^. The frequency change rate of the patch antenna was calculated from the difference between the resonant frequency when the dielectric constant was 2.19 and the resonance frequency when the dielectric constant was changed to 2.43. In Figure 3b, the frequency change hardly occurs at the point where the area is less than about (*λ*_e_/4)^2^ = 100 mm^2^, whereas the frequency change rate increases in proportion to the area as the area of the liquid crystal increases through the (*λ*_e_/4)^2^ point. As the area of the liquid crystal passes the 400 mm^2^ corresponding to the patch antenna area, it can be seen that the increase is slowed and eventually converges to a constant value. Here, *λ*_e_ means the effective wavelength considering the dielectric constant and corresponds to the length of about 40 mm.

**Figure 4:**
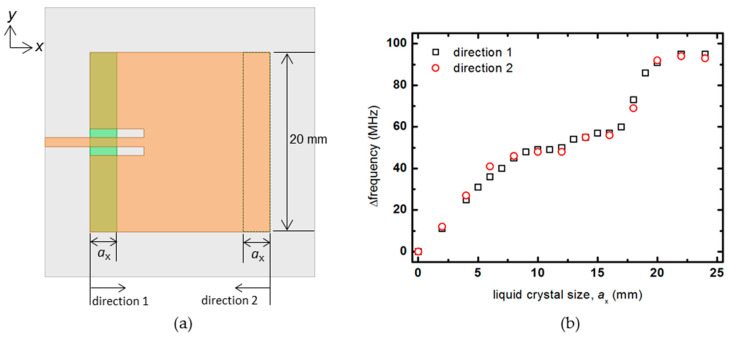
(**a**) Schematic diagram of the patch antenna that increases the size of the liquid crystal in the *x*-axis, and (**b**) the amount of frequency change.

Next, the size of the liquid crystal in the *y*-direction is fixed at 20 mm (*λ*_e_/2), and the rate of frequency change of the patch antenna is investigated by increasing the size in the *x*-direction only. Here, as shown in Figure 4, the results are confirmed by increasing the liquid crystal size in two opposite directions, direction 1 and direction 2, respectively. Similar results are obtained for both directions. While the size of *a*_x_ increases from 0 at the beginning, the frequency change rate linearly increases according to the change in the dielectric constant of the liquid crystal. On the other hand, the frequency change rate stays at 50 MHz while changing from about 8 to 12 mm. As *a*_x_ increases to 12 mm or more, the frequency change rate increases again, and when the size of the liquid crystal exceeds the patch size of 20 mm, the frequency change rate does not increase and converges to about 100 MHz. In the center of the patch antenna where the null electric field forms, even if liquid crystal is injected, it does not affect the frequency change rate much.

**Figure 5:**
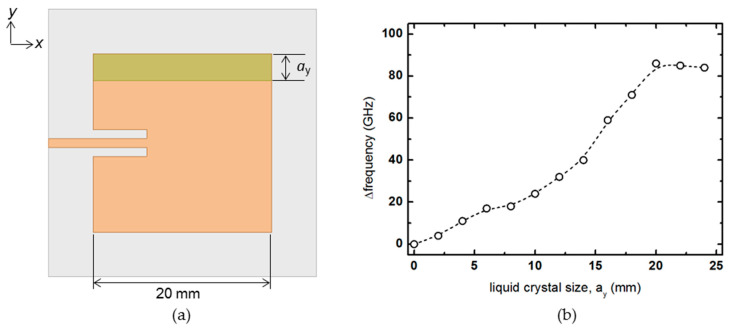
(**a**) Schematic diagram of the patch antenna that increases the size of the liquid crystal in the *y*-axis, and (**b**) the amount of frequency change.

The size of the liquid crystal in the *x*-direction is fixed at 20 mm, and the rate of frequency change is analyzed while increasing the size in the *y*-direction, as shown in Figure 5. Unlike the increase in the *x*-direction, as the size of *a*_y_ increased, the tendency is monotonically increased. However, when the *a*_y_ becomes more extensive than the patch antenna size of 20 mm, the frequency change rate becomes constant.

**Figure 6:**
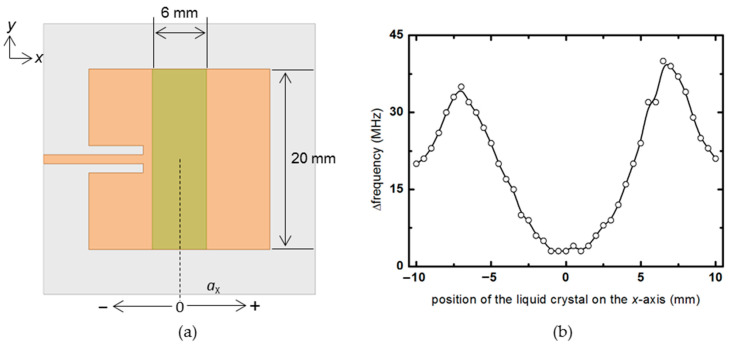
(**a**) Top view of the patch antenna showing the liquid crystal moving in the *x*-axis. (**b**) Frequency change rate according to the position of the liquid crystal in the *x*-direction.

**Figure 7:**
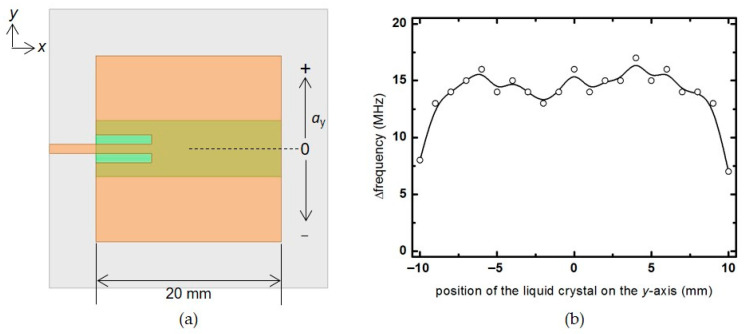
(**a**) Top view of the patch antenna showing the liquid crystal moving in the *y*-axis. (**b**) Frequency change rate according to the position of the liquid crystal in the *y*-direction.

In Figure 6 and Figure 7, the liquid crystal area is fixed, and the frequency change rate is checked while moving the position where the liquid crystal is injected. One side of the liquid crystal is set to 20 mm, which is the length of one side of the patch antenna, and the other side is fixed at 6 mm so that a certain degree of frequency change can be seen without exceeding the size of *λ*_e_/4. First, a 6 × 20 mm^2^ liquid crystal is placed parallel to the *y*-axis in Figure 6a, and the frequency change rate is checked while moving on the *x*-axis, which is the wave propagation direction. In the graph of Figure 6b, when a 6 × 20 mm^2^ liquid crystal is located near the zero points, which is the center of the antenna, it shows a slight change of less than 5 MHz, and when the liquid crystal moves near ±7 mm near both ends of the patch, it provides the maximum change rate of about 40 MHz. When the liquid crystal leaves both ends, the rate of frequency change decreases again. This simulation confirms that the change in the dielectric constant of the liquid crystal significantly affects the frequency change amount of the patch antenna when it is at both ends of the patch antenna. However, it has little effect when it is at the center.

A 6 × 20 mm^2^ liquid crystal is placed parallel to the *x*-axis, and the frequency change rate of the patch antenna is checked while moving in the *y*-axis direction in Figure 7a. In this simulation, a constant frequency change rate of about 15 MHz is shown at most locations, except for ±10 mm, where the liquid crystal deviates slightly from the area of the patch antenna. Since the electric field distribution is almost the same in the *y*-direction, when the liquid crystal with the same size is moved, the frequency change rate is uniformly displayed.

**Figure 8:**
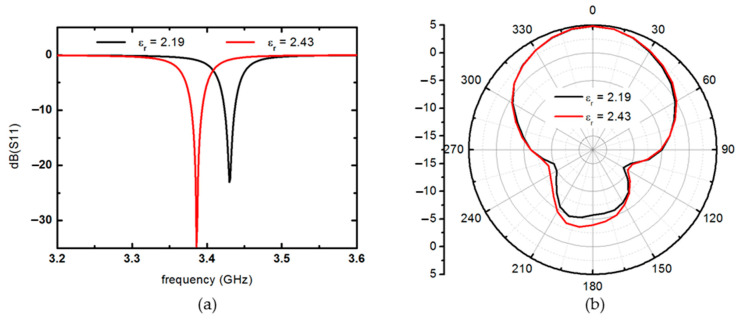
(**a**) dB(S11) examples and (**b**) E-plane radiation patterns of the patch antenna when dielectric constant changes from ε_∥_ to ε_⊥_.

As a result of confirming the horizontal distribution of the liquid crystal on the surface of the patch antenna, that is, the frequency variability according to the area and position of the liquid crystal, the larger the area where the liquid crystal is distributed, the higher the frequency variability. However, if the same amount of liquid crystal is injected, the frequency variability can be increased if the liquid crystal is injected near both ends of the patch in the *x*-direction rather than the central portion of the patch antenna. A liquid crystal having a volume of 6 mm in the *x*-direction, 20 mm in the *y*-direction, and 0.1 mm in thickness is placed at the end of the patch antenna parallel to the *y*-axis direction, and then a simulation is performed to predict the dB(S11) and gain patterns of the antenna. At this time, the tan δ of the liquid crystal is used as an estimated value of 0.01. As a result of confirming the change in the resonant frequency of the patch antenna while changing the permittivity from ε_∥_ to ε_⊥_, it can be seen that it decreases by about 40 MHz from 3.43 to 3.39 GHz, as shown in Figure 8a. In addition, the E-plane radiation patterns of the antenna appear similar to that of a typical patch antenna, as shown in Figure 8b. The peak gain is about 4.8 dBi, and the radiation efficiency is estimated to be about 73%.

### 3.2. Analysis of Optimal Liquid Crystal Thickness in Patch Antenna Substrate

Structurally, the spacing between the top metallic patch and the bottom ground plane has a dominant effect on the antenna radiation property. That is, as the substrate thickness increases, the frequency bandwidth of the patch antenna increases. Therefore, in this chapter, we analyze how the frequency change rate according to the vertical profile of the patch antenna substrate varies.

**Figure 9:**
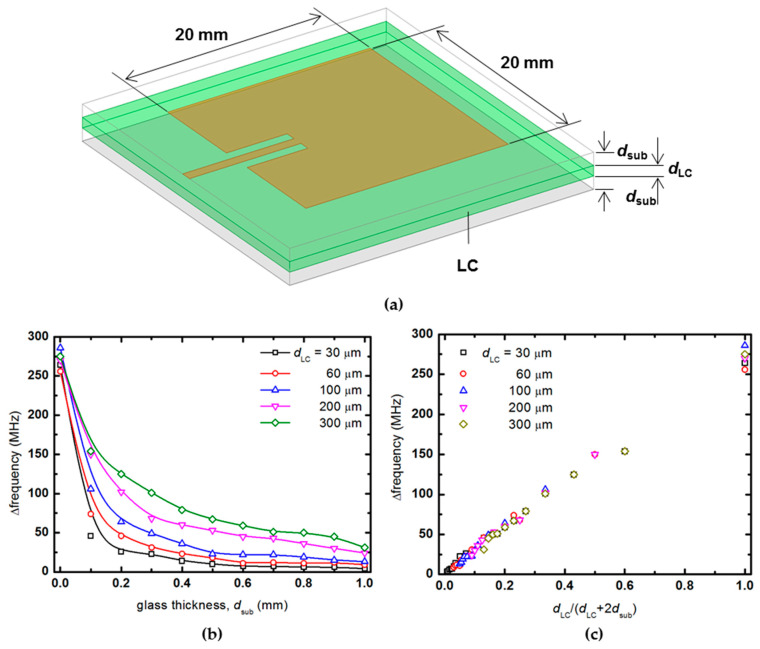
(**a**) Patch antenna substrates to investigate liquid crystal effects and frequency change according to (**b**) the absolute value change in the thickness of liquid crystal and glass and (**c**) the change in the ratio of the liquid crystal cell gap to the total thickness.

The patch antenna is fabricated by patterning metal on a printed circuit board (PCB) substrate such as glass. At this time, the top substrate and the bottom substrate are separated to inject a liquid crystal between the substrates of the patch antenna. According to the ratio of the liquid crystal cell gap to the sum of the thickness of the glass substrate on which the top patch and the bottom ground plane are located, the frequency change rate is confirmed through simulation. The cell gap of the liquid crystal is set to *d*_LC,_ and the thickness of the glass substrate was set to *d*_sub_. While changing the values of *d*_LC_ and *d*_sub_, the frequency change rate of the antenna according to the change of the dielectric constant of the liquid crystal is obtained, as shown in Figure 9b.

The thickness (*d*_LC_) of the liquid crystal was changed to 30, 60, 100, 200, and 300 μm, and the frequency change rate was confirmed while increasing the thickness of the upper and lower substrates to 1000 μm, respectively. As the liquid crystal cell gap increases, the frequency change rate of the patch antenna increases. In addition, when the cell gap is fixed, the frequency change rate decreases as the thickness of the glass with a dielectric constant increases. In Figure 9c, the frequency change rate is redrawn as the ratio of the thickness of the liquid crystal layer to the thickness of the entire substrate, not the absolute value of the thickness. Regardless of the absolute value of the liquid crystal cell gap, it can be seen that if the ratio of the liquid crystal layer to the total substrate thickness is constant, the frequency change rate is almost the same. For example, suppose the ratio of the thickness occupied by the liquid crystal layer compared to the total thickness of the antenna substrate is constant at 0.2. In that case, a frequency change rate of about 60 MHz can be secured regardless of whether the liquid crystal layer used is 100 μm or 300 μm. Therefore, in order to obtain a desired frequency change rate, it can be secured by appropriately adjusting the overall thickness rather than using an unconditionally thick expensive liquid crystal.

### 3.3. Liquid Crystal Patch Antenna Performance Enhancement Technique

Next, we propose an idea for maximizing the previously analyzed frequency change rate and securing the bandwidth of the patch antenna. First of all, when fabricating a patch antenna based on a liquid crystal, an alignment layer must be laid on the upper and lower surfaces of the liquid crystal to secure the dielectric anisotropy of the liquid crystal sufficiently. At this time, the alignment force of the alignment layer is rapidly weakened at a thickness of 100 μm or more [8]. Therefore, it does not make much sense to use a liquid crystal layer with a thickness of 100 μm or more when manufacturing a liquid crystal patch antenna.

**Figure 10:**
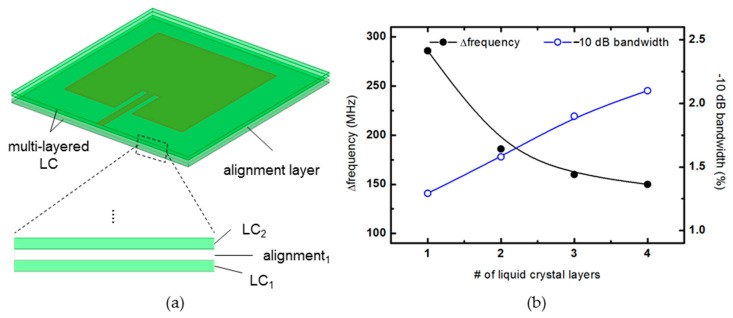
(**a**) 3D view and detail of the multi-layered LC substrate. (**b**) Frequency change rate and −10 dB bandwidth according to the number of liquid crystal layers.

On the other hand, in terms of antenna radiation performance, since the frequency bandwidth increases as the thickness of the substrate increases, it is advantageous to make the thickness of the patch antenna substrate as thick as possible unless unwanted modes occur. Since a patch antenna operating in the several GHz bands can secure a reasonable bandwidth when using a substrate with a thickness of several hundred μm, this paper proposes a method to increase the thickness of the antenna substrate while maintaining the alignment force. As shown in Figure 10a, this is a method to increase the thickness of the entire substrate by additionally inserting an alignment layer for each 100 μm-thick liquid crystal layer. In this case, the thickness of the liquid crystal layer does not exceed 100 μm, and the overall thickness of the antenna substrate can be increased. However, as previously analyzed, since the substrate used as the alignment layer is additionally inserted, the ratio of the thickness occupied by the liquid crystal layer among the thicknesses of the entire substrate is reduced.

Figure 10b shows a graph comparing the frequency bandwidth and frequency variation of the antenna while increasing the alignment layer inserted in the middle one by one. The thickness of one liquid crystal layer is set to 100 μm, and the thickness of the inserted alignment layer is also set to 100 μm. For example, when one alignment layer is inserted, the liquid crystal layer becomes two, one above and one below, so the ratio of the liquid crystal cell gap to the total thickness is 2/3. When two alignment layers are inserted, the liquid crystal layers are divided into three, and the ratio of the liquid crystal cell gap to the total substrate thickness is 3/5. As expected, as the number of 100 μm-thick liquid crystal layers inserted, the total thickness of the antenna substrate increased, increasing the frequency bandwidth, while the rate of frequency change decreased. In case of the thickness of the layers inserted for the LC alignment to be less than 100 μm, it is possible to suppress a decrease in frequency change rate due to an increase in the active layer ratio (∑*d*_LC_/(∑(*d*_LC_ + *d*_sub_))). Therefore, when designing a liquid crystal patch antenna, it is expected to be able to design it by appropriately selecting the number of liquid crystal layers in consideration of the desired bandwidth and frequency change rate.

## 4. Conclusions

A method of effectively designing a patch antenna for variable frequencies using the dielectric anisotropy of liquid crystal has been described. By analyzing the primary electric-field distribution of the patch antenna and the radiation performance of the patch antenna according to the substrate thickness, a study is conducted on where it is effective to inject a liquid crystal to maximize the frequency variation of the patch antenna. By proposing a method of maximizing the amount of frequency change using a minimum amount of liquid crystal, a technology for efficient dispersing of expensive liquid crystals on the substrate is secured. The development of a more practical liquid crystal smart antenna can be accelerated when the remaining tasks include developing a liquid crystal material optimized for the microwave band, the optimization of the structures for low voltage driving, and the rapid response to voltage changes can be solved.

## Data Availability

The data presented in this study are available on request from the corresponding author.
